# Assessment of menstrual health status and evolution through mobile apps for fertility awareness

**DOI:** 10.1038/s41746-019-0139-4

**Published:** 2019-07-16

**Authors:** Laura Symul, Katarzyna Wac, Paula Hillard, Marcel Salathé

**Affiliations:** 1 Department of Surgery, Stanford School of Medicine, Stanford University, 300 Pasteur Dr., Stanford, CA 94305-5317 USA; 2 Digital Epidemiology Lab, Global Health Institute, School of Life Sciences, École Polytechnique Fédérale de Lausanne (EPFL), Campus Biotech, Chemin des mines 9, 1202 Geneva, Switzerland; 3 Quality of Life Technologies lab, Institute of Services Science, Center for Informatics, University of Geneva, CUI Battelle bat A, Route de Drize 7, 1227 Carouge, Switzerland; 4 DIKU, University of Copenhagen, Copenhagen, Denmark; 5 Department of Obstetrics & Gynecology, Stanford School of Medicine, Stanford University, 300 Pasteur Dr. HH333, Stanford, CA 94305-5317 USA

**Keywords:** Reproductive signs and symptoms, Epidemiology, Computational models

## Abstract

For most women of reproductive age, assessing menstrual health and fertility typically involves regular visits to a gynecologist or another clinician. While these evaluations provide critical information on an individual’s reproductive health status, they typically rely on memory-based self-reports, and the results are rarely, if ever, assessed at the population level. In recent years, mobile apps for menstrual tracking have become very popular, allowing us to evaluate the reliability and tracking frequency of millions of self-observations, thereby providing an unparalleled view, both in detail and scale, on menstrual health and its evolution for large populations. In particular, the primary aim of this study was to describe the tracking behavior of the app users and their overall observation patterns in an effort to understand if they were consistent with previous small-scale medical studies. The secondary aim was to investigate whether their precision allowed the detection and estimation of ovulation timing, which is critical for reproductive and menstrual health. Retrospective self-observation data were acquired from two mobile apps dedicated to the application of the sympto-thermal fertility awareness method, resulting in a dataset of more than 30 million days of observations from over 2.7 million cycles for two hundred thousand users. The analysis of the data showed that up to 40% of the cycles in which users were seeking pregnancy had recordings every single day. With a modeling approach using Hidden Markov Models to describe the collected data and estimate ovulation timing, it was found that follicular phases average duration and range were larger than previously reported, with only 24% of ovulations occurring at cycle days 14 to 15, while the luteal phase duration and range were in line with previous reports, although short luteal phases (10 days or less) were more frequently observed (in up to 20% of cycles). The digital epidemiology approach presented here can help to lead to a better understanding of menstrual health and its connection to women’s health overall, which has historically been severely understudied.

## Introduction

**Fig. 1:**
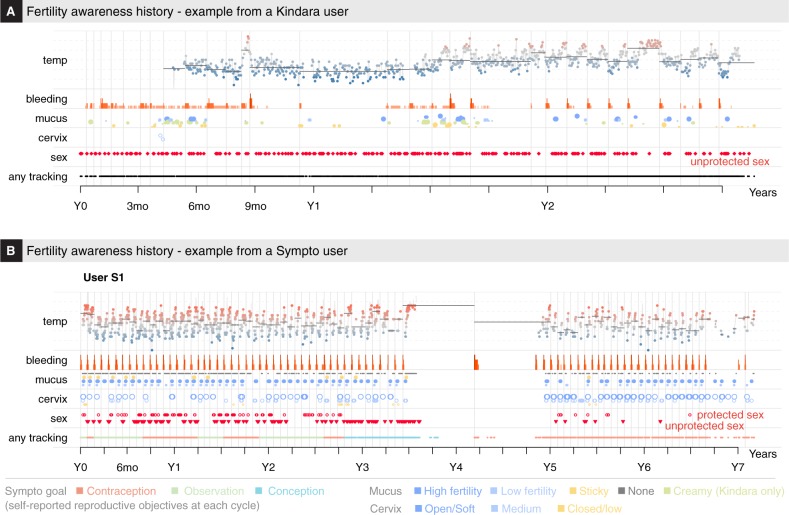
Menstrual history of two app users. Menstrual history of two long term Kindara **a** and Sympto **b** users. Time is shown in years as relative to the first observation of each user. Kindara user is seeking to achieve pregnancy and shows a long anovulatory episode during which her overall temperature is lower. She returns to more regular, ovulatory cycles in her last year of tracking, as indicated by the bleeding frequency and the temperature profiles. The Sympto user has used the app to avoid pregnancy and observe her cycle for almost 3 years, before trying to conceive, which she likely achieves after 9 cycles (her reported cycle-specific reproductive objective switches from “contraception” to “conception”—line “any tracking” at the bottom). Nine months later, the user reports bleeding, which likely indicates post-partum bleeding (lochia). After another 9 months, probably as she stops breastfeeding, she logs menstrual observations and returns to using the app to avoid pregnancy

**Fig. 2:**
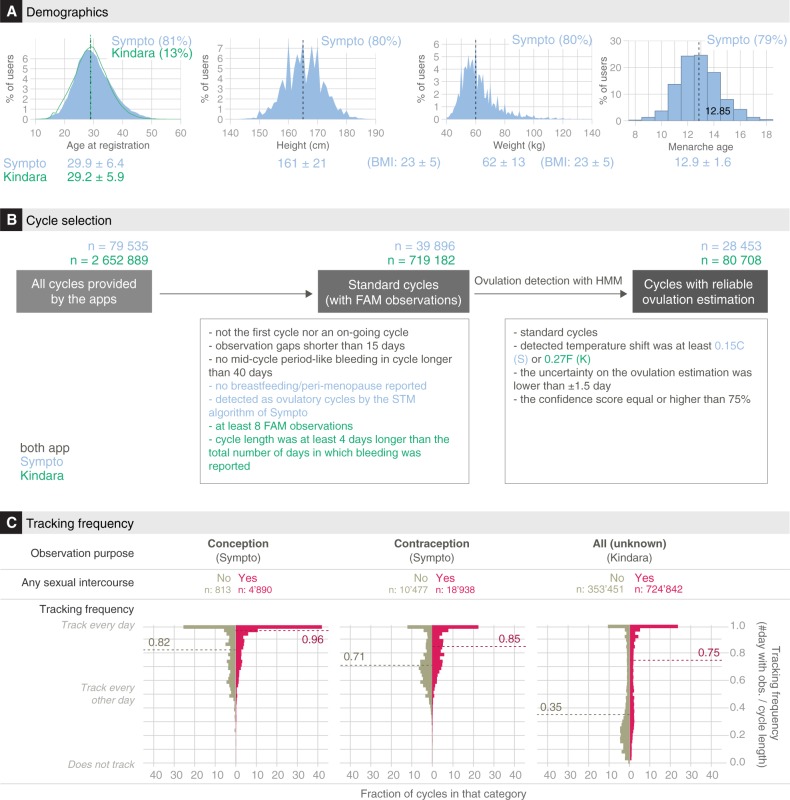
Demographics and tracking behavior of users. **a** Users’ age at registration (left), reported height (middle-left), weight (middle-right) and menarche age of users (right). The percentages on the top-right corner of each histogram is the fraction of users for which the information was available in the dataset. The lower line provides the mean ± standard deviation of the corresponding variables as well as of the Sympto users’ BMI, calculated as their weight divided by their square height (in m). **b** Cycle selection flowchart. Methods provide extensive description of the inclusion/exclusion criteria. Standard cycles are finished, complete cycles, typical of a non-pregnant, non-peri-menopausal, non-nursing user, that have at least 8 days with FAM observations (Kindara) or that are detected as ovulatory cycles according to the Sympto implementation of the STM rules. Cycles with reliable ovulation estimation are cycles for which the ovulation day could be reliably estimated by the HMM framework developed for this study (Methods). **c** Cycle-specific tracking frequencies (top: Sympto, bottom: Kindara). 39,896 (Sympto) +719,182 (Kindara) standard cycles were used (Methods). Dashed lines indicate median values

A broad diversity of fertility awareness methods (FAMs) has been developed in the past century,^1,2^ primarily designed to help couples manage fertility and family planning. Modern methods developed in the last quarter of the twentieth century take advantage of the precise description of menstrual variation of the basal body temperature (BBT) or waking temperature, taken with a thermometer with a 0.01 °C or 0.5 °F precision, cervical mucus quality and quantity, vaginal sensation, and cervical position.^3–6^ These methods have defined a set of rules that allows the identification of the fertile window around ovulation, so that couples can adapt their sexual behavior according to their reproductive objectives.^7–9^ The sympto-thermal method, which combines BBT and cervical mucus observations, is arguably amongst the most reliable FAM for family planning.^1,2,4,10^ Recently, a number of mobile apps have been developed by private organizations to facilitate FAM tracking. Some of these apps provide their users with automatized interpretation with regard to the opening and closing of the fertility window.^11^ Over the past few years, an increasing number of women, estimated at over 200 million in 2016,^12^ have started using these apps, contributing to the accumulation of menstrual-related data (Fig. 1) from a diverse population of users at different stage of life (Fig. 2a; Table 1, see Methods).

**Table 1:** Number of observations, cycles and users

App	Dataset	Total # of users	Total # of cycles	Total # of days of observations	Avg # of cycles per users	Fraction of full dataset (wrt # of users) (%)	Fraction of full dataset (wrt # of cycles) (%)	Fraction of full dataset (wrt # of observations) (%)
Sympto	Full dataset	13,674	79,535	1,622,270	5.82			
	Standard cycles	5,860	39,896	949,358	6.81	43	50	59
	Cycles with reliable ovulation estimation	5,116	28,453	670,989	5.56	37	36	41
Kindara	Full dataset	199,293	2,652,889	32,053,183	13.31			
	Standard cycles	125,170	719,182	15,987,512	5.75	63	27	50
	Cycles with reliable ovulation estimation	27,378	80,708	2,248,666	2.95	14	3	7
Total	Full dataset	212,967	2,732,424	33,675,453	12.83			
	Standard cycles	131,030	759,078	16,936,870	5.79	62	28	50
	Cycles with reliable ovulation estimation	32,494	109,161	2,919,655	3.36	15	4	9

Number of users, cycles and days of observations. In a single day, a user can log up to 7 observations, i.e., one in each of the tracking categories available to users, see Table 2

A few studies have evaluated some of these apps in terms of user experience or the accuracy of the scientific information provided to their users^13,14^ or regarding their ability to accurately indicate the opening and closing of the fertile window.^11,15^ In 2016, Moglia et al. and Duane et al. evaluated that few applications were accurate, both in terms of cycle length prediction^13^ or in terms of fertility window estimation,^11^ and that few apps were endorsed by medical professionals^13^ or relied on evidence-based FAM.^11^ These studies provide app rankings according to their usability and accuracy of the medical information provided by the apps,^13^ their ability to support the use of FAM to avoid pregnancy^11^ or to increase conception chances.^15^ Other studies^16–18^ have evaluated the contraceptive efficacy of the app Natural Cycles; this app based on a proprietary algorithm only takes body temperature into account.^16–18^ These studies were authored by at least one of the app founders and did not provide a description of the tracked data. They assessed the typical-use and perfect-use Pearl Index of their app based on retrospective data first (perfect-use: 0.5, typical-use: 7) then designed prospective study on a larger population which corrected their typical-use Pearl Index to 6.9 and their perfect-use to 1.0.^16–18^ They also report a discontinuation rate of 54% after 12 months.^17^ In the last two years, only a few studies have used datasets from women’s health applications, such as Clue, to test medical hypotheses or to develop analysis frameworks suited for menstrual cycle analyses. Notably, a 2018 study by Alvergne et al. suggests that negative premenstrual experiences might be aggravated by the presence of undiagnosed sexually transmitted infections.^19^ Recently, studies have used similar data, including menses reports but no fertility awareness data, to develop novel machine learning methods suited to study rhythmic human behaviors^20^ or predict pregnancy.^21^ The latest study compares several models, including neural networks, to predict pregnancy chances in an on-going cycle. The predictive power was relatively low and the method was not suited for irregular cycles but was shown to be able to recover an average fertile window.^21^

Fertility awareness body signs, as tracked easily via accessible mobile applications, have not yet been extensively described or studied and it is unclear how app users are reporting these signs, as well as whether the reported observations are consistent with the conclusions of previous smaller-scale medical studies.^6,22,23^ Moreover, there are no statistical frameworks to detect ovulation from these self-tracked data, which would be useful to leverage the potential of these data to study fertility, accurately predict pregnancy chances and to overall evaluate the potential impact of fluctuating hormones on the course of chronic diseases.^24^

To fill these gaps, the present study pursued two main objectives. The first aim of this study was to describe the typical users, their tracking behavior and to provide an overview of the observations they logged in the apps. The second aim was to provide a statistical framework for the estimation of ovulation time from these self-reported data, which allowed for the comparison of cycle length and ovulation time with previously reported values from medical, non-digital, studies. We used datasets from two independent mobile phone apps (Sympto and Kindara, Methods) comprising 1.6 and 32 million observations, respectively.

## Results

### Users demographics: the typical FAM app user is 30 ± 6, has a healthy BMI (23 ± 5), and lives in a European or North American country

The two apps target different populations. Users of these two apps are found in over 150 countries, covering 5 continents, but the vast majority of them are located in Europe and in the Americas. Most Kindara users are based in the US and are trying to achieve pregnancy, while Sympto users mainly reside in Europe and use the app primarily to avoid pregnancy. User ages span the reproductive life of women, from the onset of their sexual activity to menopause, with an over-representation of users in their late 20s and early 30s (Fig. 2a, left). For some users, additional information is available, including their birth year, and, for Sympto users only, their reported weight, height and age at menarche (Fig. 2a).

The height and weight distribution of Sympto users (Fig. 2a, top and bottom right, data not available for Kindara users) shows median values of 60 kg (132lbs) and 165 cm (5 ft 5in). Both distributions present peaks at round values such as 160 or 165 cm indicating that users often report approximate values (for example, 160 cm rather than 159 or 161 cm). This has been observed in previous studies using self-reported values and these mild inaccuracies of self-reported values have usually been found to only slightly affect the overall distributions.^25^ The median BMI of Sympto users is around 20, which is considered healthy for women (Supplementary Fig. 1C). Information such as users’ level of education, marital or social status, parity or particular health conditions are unknown.

### Users log their observations at a higher frequency when they also log sexual intercourses

The tracking behavior of regular FAM users during their usual cycles, which here are referred to as “standard cycles” (Fig. 2b, Methods) is highly variable and depends on the family planning objectives of the users (Fig. 2c). For an idealized ~28-day cycle, FAM-relevant body signs need to be recorded for at least 8–12 days of each cycle to detect the changes related to ovulation. This represents a tracking frequency of at least ~43%. However, most users using the apps for their FAM tracking report their observations for over 16 days per cycle. In cycles where users choose to record sexual intercourse (65% (S)–75% (K) of standard cycles), tracking frequency is increased, with over 40% of cycles being tracked every single day when seeking pregnancy (Fig. 2c and Supplementary Fig. 1D), sometimes for several months or years in a row (Fig. 1).

Tracking frequencies varied between the two apps (Fig. 2c), partly in relationship to the design of the apps; Kindara doesn’t provide user interpretation of the fertility window allowing for sporadic tracking, whereas missing data in Sympto precludes an accurate fertility assessment.

### Reported fertility awareness body signs exhibit temporal patterns at the user population level

**Fig. 3:**
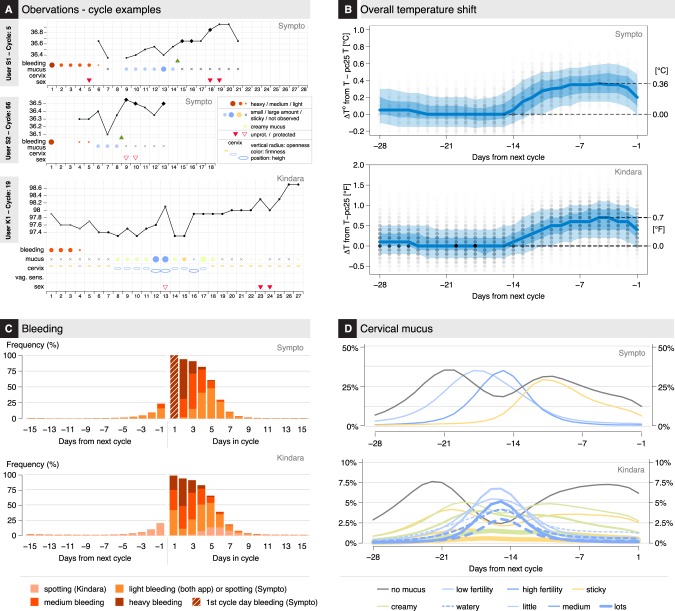
User observations overview. **a** Examples of observations: the 5th tracked cycle (top) and 66th cycle (middle) cycle of two different Sympto users. Observations of the 19th cycle (bottom) of a Kindara user. **b** ΔBBT (variation from the 25% percentile of temperature in this cycle) values are shown on each day of the cycle, from the end of the cycle. Opacity of the dots reflects the number of observations. The median value: thick blue line. 10, 25, 75, and 90 percentiles of ΔBBT: translucent blue bands. **c** Frequency of bleeding observations, for the end (left) and beginning (right) of cycles. The Sympto app only starts a new cycle on the first recording of heavy bleeding (score 3/3, dark red) after a post-ovulatory infertile phase, thus all cycles present heavy bleeding at the start of the cycle (hashed dark red bar). **d** Frequency of cervical mucus observations from the end of cycles (top: S, bottom: K). (Kindara) Little quantity of watery mucus (dashed line) and little or medium quantity of egg-white like mucus (solid line) are considered as “low fertility” mucus (light blue) while large quantities of egg-white like and medium or large quantities of watery mucus are considered as “high fertility” mucus (dark blue) (B-D) 39,896 (S) +719,182 (K) *standard cycles* were used (Methods)

Confident that users regularly logged observations (Fig. 2c) during standard cycles, we sought to characterize general patterns in the observations and frequency of the different FAM body signs and investigate whether they were consistent with previous studies.^5,6,9,26,27^ As cycle durations vary by several days, as illustrated in Fig. 3a, and given that the duration of the luteal phase (after ovulation) has been shown to vary less than the follicular phase (before ovulation),^28,29^ ovulation-related observations (BBT, mucus, cervix, vaginal sensation) are shown from the end of each cycle (Fig. 3b–d and Supplementary Fig. 2). A clear shift of about 0.36 °C/0.7 °F in BBT between the mid-follicular phase and the mid-luteal phase is observed (Fig. 3b and Supplementary Fig. 2A), consistent with previous observations on a cohort of much smaller size.^26^ BBT showed a decrease at the end of the cycle, as light bleeding or spotting was reported (Fig. 3b, c).

In an ovulatory cycle, it is well established that cervical mucus is produced in higher quantity and with a higher stretchiness in the days leading up to ovulation,^5,6,9,27^ which seems to be observed by users tracking their cervical mucus (85–90% (S) and 40–45% (K) of cycles) (Fig. 3d).

### Estimation of ovulation day from fertility awareness body-signs

**Fig. 4:**
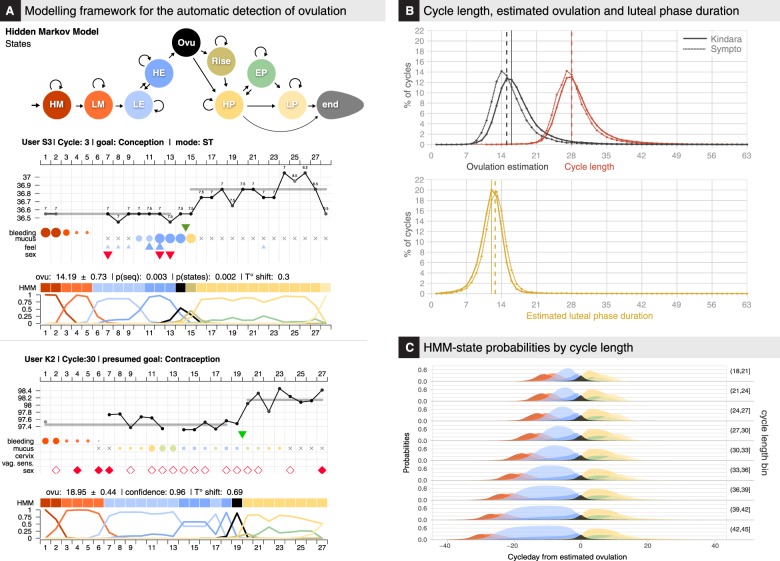
Modeling framework for the estimation of ovulation and menstrual states. **a** Modeling framework for the estimation of ovulation timing. (Top) Schematics of the 10-states HMM which discretizes the menstrual hormonal events (HM, heavy menses; LM, light menses; LE, low estrogen; HE, high estrogen; Ovu, ovulation; Rise, progesterone/BTT Rise; HP, high progesterone; EP, estrogen peak in luteal phase; LP, low progesterone). Arrows indicate possible state-transition; arrow thickness is not representative of actual transition probabilities (Methods). (Bottom) Examples of menstrual state estimation for the 2rd and 3rd cycle of 2 users. (Top of each chart) Original user observations as in Fig. 2a. (Middle of each chart) Colored squares HMM-labeled line) represent the most likely sequence of HMM states given the observations (Methods). (Bottom of each chart) Normalized probabilities of each state on each day of the cycle (Methods). **b** (Top) Cycle length and estimated ovulation day. (Bottom) Luteal phase duration, computed as the number of days between the ovulation day (excluded) and the 1st day of the next cycle (excluded). Vertical lines indicate median values. 80,708 (K) +24,119 (S) cycles with reliable ovulation estimation were used (Methods). **c** Average estimated state probabilities by cycle-day counting from estimated ovulation aggregated by total cycle length (in bins of 3 units) for all cycles with reliable ovulation estimation

Previous studies have shown that the combination of BBT and cervical mucus variations were reliable, although not perfect, proxies for the detection of ovulation.^8,23,27,30^ We therefore decided to define a mathematical framework (HMM) to derive an estimate of the most likely day of ovulation with reliability indicators to reflect the uncertainty of conflicting or unexpected observation patterns (Fig. 4a and Supplementary Figs 3, 4, 6, Methods). Missing temperature records have been found to alter the precision of the ovulation estimation to a slightly greater extent than missing cervical mucus reports (Supplementary Fig. 6D, Supplementary Material).

These estimations allowed the comparison, for cycles with reliable ovulation estimation (109,161 cycles, Methods), of the cycle length distribution to those of estimated day of ovulation and of the duration of the luteal phase (i.e., post-ovulation) (Fig. 4b). Cycle length distribution is asymmetrical around the typical 27 to 28 days, with a heavy tail on longer cycles. Similarly, the distribution of the follicular (i.e., prior to ovulation) phase duration (or ovulation time) is asymmetrical as well, with a median value of 16 days, and 90% of ovulations occurring between day 10 and day 24. Only ~24% of ovulations occurred on days 14 to 15 of the cycle.

Luteal phase duration distribution, which is also asymmetrical, presents however a skew for smaller values and a smaller standard deviation (Fig. 4b, c and Supplementary Fig. 4BC). Median values were 12 (K) and 13 (S) days, which is in line with a previous study that used fertility monitors^31^ but shorter than values reported in studies that used luteinizing hormone (LH) peak for timing of ovulation (14 days).^29^ About 35% of cycles have a luteal phase duration of 12–13 days, while ~20% of cycles had a luteal phase duration smaller than or equal to 10 days, which represents a higher proportion than reported in a previous epidemiological study (4.5%).^29^

Overall, the comparison with previous studies of the cycle phases duration and range shows that the follicular phase and the whole cycle length have higher mean values and larger ranges than what was previously observed, while the luteal phase duration and range was closer to those found in previous studies^28,29,31–33^ (Supplementary Fig. 5).

## Discussion

This study’s goal was to describe and explore the suitability of datasets collected through two mobile applications (Kindara and Sympto) supporting Fertility Awareness Method (FAM) tracking for the assessment of menstrual health in general, both at the individual level and at the population level. The primary aim was to provide health practitioners with an overview of how and what FAM app users voluntarily track on these apps. Many, if not most clinicians are unfamiliar with the specifics of health-related apps, and thus the information from this study may provide clinically helpful information. The secondary aim was to propose a mathematical framework to estimate the underlying hormonal states and most likely day of ovulation from FAM observation. This allowed a comparison of the duration of the menstrual cycle phases from the present digital study with reported values from previous clinical studies.

The typical FAM app user is about 30 years old, lives in a western country (in Europe or Northern America) and has a healthy BMI. The height, weight and BMI ranges reported by Sympto users are similar to those reported for the French population,^34^ which is where most Sympto users are located. Thus, to the extent that these users differ from the general population, our results may be more or less generalizable to other populations.

The tracking frequency of users that utilize the apps for FAM tracking, is on average higher than the minimum required to detect changes associated with ovulation. In particular, if users rely on the app for their family planning, i.e. if they log sexual intercourses (protected or unprotected), the tracking frequency is increased, with up to 40% of cycles having recordings every single day when the user’s objective is to achieve pregnancy.

The reported FAM observations (BBT, cervical mucus changes, cervix openness, etc.) are overall aligned with expected patterns of FAM-related body signs, showing that these apps enable hundreds of thousands of users across Europe and North America to follow their fertility and ovulation patterns. Temperature is found to increase by 0.36 °C/0.7 °F after ovulation, while cervical mucus is reported more abundantly, stretchy and transparent in the days around ovulation, consistent with previous description of these body signs variations. The aggregated patterns of the reported menstrual body-signs are in good agreement between the two applications despite different app design, user experience and targeted populations (Methods).

Individual cycles often present noisy profiles, and missing data are a frequent concern. To partly alleviate these issues, the mathematical framework (HMM) used in this study discretizes the menstrual cycle in independent successive biologically-relevant states and allows the estimation of ovulation timing along with uncertainty indicators. The variation range in the ovulation time and in the luteal phase duration was found to be larger than previously described in other studies^29,31,32,35^ that relied on much smaller populations but that used biomarkers which offer a greater precision for the estimation of ovulation time. The larger observed mean and range of the follicular phase and of the cycle length can partially be explained by the differences in the data inclusion/exclusion criteria—for example, some previous studies excluded long cycles (Supplementary Table 9)—and by the ovulation estimation methods, but also probably by the fact that this study uses cycles from a much larger population and is thus able to capture a higher diversity of menstrual patterns. Interestingly, the cycle phases distributions were slightly different when considering the data from the two apps. These differences might be due to biases found in the user population, especially for users seeking pregnancy that could be at higher risk of sub-fertility if assumed that they start tracking after they have already tried to get pregnant for several months (Supplementary Fig. 4C); however, these data on user behaviors around fertility seeking are not available for Kindara users.

The strength of this study lies in the scale and precision of the datasets, as a variety of fertility patterns are captured, and as users track the evolution of their cycles at a high frequency over long intervals of time. It also provides a non-proprietary and replicable mathematical method to infer biological states, and in particular to estimate the timing of ovulation, from fertility awareness self-tracked data. The most obvious potential limitation of this study comes from the origin of these retrospective data: a self-selected possibly biased population, limited medical and general information on users, irregular observation patterns and little control on assessing the validity of the observations, in particular with regard to cervical mucus tracking. While the tracking frequency limitation can be alleviated through strict selection of users and cycles (Methods), all other limiting factors might have introduced biases in the present analysis. Prospective studies on selected cohorts with appropriate follow-up and information provided to users will provide higher quality data, which could then be used for comparison.

While this study does not assess the benefits for users to use tracking apps compared to relying on their memory or charting their cycles on paper or in their personal calendars, it provides clinicians and (digital) epidemiologists with an overview of the expected tracking behaviors and body-signs patterns, so that they can evaluate the suitability and benefits of digital self-tracking for their clinical practice or for the design of prospective studies. Based on the current findings, it appears that digital self-tracking of FAM-related body signs could provide a more accessible, although less precise, means to evaluate the status and evolution of menstrual health than traditional medical monitoring which requires frequent office visits for ultrasounds or hormonal testing from blood or disposable urinary tests. The self-tracked observations presented here require only a standard thermometer with a 0.05 °C resolution, and simplified versions of these apps are provided for free. Digital self-tracking, compared to paper-based tracking or memory-relying surveys, supplies standardized records and scalable collection methods. Typically, digital self-tracking of fertility-awareness body signs offers an interesting option for clinicians or researchers interested in changes of a variable of interest (for example level of pain or occurrence of a given symptom) across the menstrual cycle, or in the overall changes in menstrual rhythmicity. For investigations requiring a precise assessment of hormonal levels or ovulation timing, additional tests would be necessary until the accuracy and precision of methods using FAM digital records can be established.

The long term and yet very precise recordings presented in this study support the idea that the menstrual cycle, like other biological rhythms, is a vital sign whose variations inform about overall health status.^36,37^ The digital epidemiology approach,^38^ where patients collect data themselves through digital means, can in this context represent a powerful method to investigate menstrual health and its connection to women’s health at the population level^33^ in a field that has historically been severely understudied.^39^

We foresee that future studies will use self-tracked data to quantify infertility or daily pregnancy chances based on reported FAM body signs and user’s history. Models could also be established to investigate potential sub-fertility causes (anovulation, recurrent early pregnancy losses, etc.) based on the fertility signs and user’s sexual behavior. More generally, such data and tracking apps, combined with tracking of other coexisting symptoms, health indicators or behavioral markers, enable the exploration of the menstrual dimension of the course of chronic diseases.^24,40^ Such studies would highly benefit from additional, sometimes already existing, tracking options in the apps such as pregnancy validation (for example reports of pregnancy tests results) or a prompt to the user to label a tracking pause such that it can reliably be differentiated from a pregnancy. Many menstrual symptoms associated with the pre-menstrual syndrome (PMS), such as mastalgia (breast pain), or disease, like migraine that can exist in a menstrual or non-menstrual form, have been shown to be associated with steroid hormones although the exact causes have not been elucidated yet.^41–46^ Future studies using self-reported occurrence, severity and frequency of such symptoms in large population and in relationship to menstrual health might allow for the investigation of associations or specific phenotypes, i.e. distinct forms of symptom expression in the population.

It is likely that users of such applications already have an increased awareness of their cycles, and this study suggests that these digitally self-tracked observations potentially present an opportunity to facilitate the dialog between patients and their clinicians, helping them to make informed decisions based on quantified indicators. The current and future development of evidence-based digital tools for menstrual health monitoring could positively impact women’s health.

## Methods

Extended Materials and methods can be found in the Supplementary Materials.

To briefly summarize the methodology used in this study: datasets were first filtered to keep cycles of users using the apps for fertility awareness purposes, i.e. to self-identify their fertility window, for at least 4 cycles. Data were then aggregated to describe the overall observation patterns. Finally, a Hidden Markov Model (HMM) was defined and used to detect ovulation time and assess the reliability of this estimation.

### Mobile phone applications and data acquisition

Two de-identified retrospective datasets were acquired from the Symptotherm foundation (www.sympto.org; Switzerland) and Kindara (www.kindara.com; US) upon receiving ethical approval from the Canton Geneva ethical commission (CCER Genève, Switzerland), study number 2017–02108. These two apps were selected as they both ranked high in a study comparing the performances of apps marketed to avoid pregnancy using FAMs,^11^ as their privacy policies specified the use of their de-identified datasets for research purposes and as their user pools were very large or diverse geographically and culturally. Sympto was released in 2008 and is available worldwide in eight languages (English, French, German, Italian, Spanish, Polish, Russian, and Bulgarian). Kindara has been released in 2012 and is available worldwide in English. Both organizations de-identified their datasets before transferring them to the authors. Both apps are available on iOS and Android platforms and are available as free (simplified) or paid apps. All features used in this study are available in the free versions of the apps. Kindara provided a random subset of their overall pool of users with at least 4 logged cycles (199 293 users, 2,652,889 cycles) while Sympto provided observations from their long-term users (at least 4 cycles tracked with the app) and from users who provided their weight, height and menarche age (13,674 users, 79,535 cycles). Both apps offer similar FAM tracking options but differ in their design and user experience (Supplementary Fig. 1AB, Table 2). A description of the datasets fields is provided in Table 2. Kindara (K) is primarily marketed to women who wish to achieve pregnancy and does not provide feedback to users in terms of the opening or closing of their fertile window. Sympto (S) is marketed as a family planning tool that can be utilized to plan or avoid a pregnancy. The Sympto app provides feedback to their users based on their observations, indicating when they are potentially fertile, very fertile or infertile. The key differences between these two apps are (i) the algorithmic- (S) vs. user- (K) interpretation of observations, (ii) the per-cycle (S) vs. per-user (K) definition of fertility goals users wish to achieve, (iii) the criteria for the onset of a new cycle, i.e., fresh bleeding after ovulation (S) vs. self-assessed or automatic, based on first day of reported bleeding (K), and (iv) the resolution at which users can report their observations (Table 2, Supplementary Material).

**Table 2:** Reported observations

Type	Sympto		Kindara	
	Unit/categories	Max precision/subcategories	Unit/categories	Max precision/subcategories
BBT	Celsius	0.05	Fahrenheit	0.01
BBT time	Daytime	1/2 h	Daytime	Minute
Questionable temp	(Not recorded)		Logical	
Mucus	NA		NA	
	No mucus		No mucus	
	Little amounts of creamy/egg-white like mucus or not very stretchable mucus		Creamy	Little/medium/lots
	Large amounts of egg-white like, watery, very stretchable mucus		Egg-white like	Little/medium/lots
			Watery	Little/medium/lots
	Sticky mucus		Sticky	Little/medium/lots
Cervix	NA		Height	Low/medium/high
	Closed, firm, low		Firmness	Firm/medium/soft
	medium		Openness	Closed/medium/open
	Open, soft, high			
Vaginal sensation	NA		NA	
	Dry		Dry	
	Wet		Dry sticky	
	Very wet		Wet moist	
			Wet lubricate	
Sex	Protected		Protected	
	Unprotected		Unprotected	
			Withdrawal	
			Insemination	

Tracking options available to users of the Sympto and Kindara app. Kindara offers more granularity and categories for reporting mucus, cervix and vaginal sensation. Provided that they primarily market users who wish to achieve pregnancy, they also offer the option to track insemination. Sympto considers withdrawal as unprotected sex and does not offer that option to their user

### Selection criteria for users and cycles

Given that these are self-tracked data, missing data is a frequent issue, and many cycles within the datasets provided by the app were not suitable for the analyses of this study. We followed an iterative approach in which we first inspected the raw datasets and identified patterns or behavior that were inconsistent with the aims of the study (for example, on-going cycles). This inspection of the datasets led to the establishment of inclusion/exclusion criteria such that cycles were filtered to remove any unfinished or uncomplete cycles or cycles in which fertility awareness body signs were not reported by the users. Resulting cycles that were kept for the analysis and the description of the reported FAM body-signs were labeled as “standard cycles” (see flowchart, Fig. 2b). Finally, the HMM was used to estimate ovulation and, for the reports of cycle length, follicular and luteal phase durations, only cycles in which ovulation could reliably be estimated were kept (Fig. 2b). Below are the inclusion/exclusion criteria for these cycle categories.

#### Standard cycles

(Sympto: 39,896 cycles; Kindara: 719,182 cycles) denote cycles of regular users of the apps in which FAM body signs have been logged. Typically, cycles with long tracking gaps or in which only the period flow was logged were excluded.

- (S&K) not the first cycle of a user nor an on-going cycle.
- (S&K) observation gaps were no longer than 15 days within a given cycle.
- (S&K) at least one FAM body sign (BBT or cervical mucus or cervix position) was recorded.
- (S&K) no mid-cycle period-like bleeding was detected when the cycle was longer than 40 days.
- (S) defined as ovulatory cycles by the STM algorithm of Sympto, i.e., in which the fertile window could be closed.
- (K) at least 8 FAM observations were reported.
- (S) no breastfeeding was reported or peri-menopause was declared.
- (K) cycle length was at least 4 days longer than the total number of days in which bleeding was reported.

#### Cycles with reliable ovulation estimation

(Sympto: 28,453 cycles; Kindara: 80,708)

Criteria summary:

- Standard cycles
- Detected temperature shift was at least 0.15 °C (S) or equivalently 0.27 °F (K) (Supplementary Material).
- The uncertainty on the ovulation estimation as provided by the HMM framework developed here was lower than ±1.5 day (see Methods below and Supplementary Material).
- The confidence score, which is related to acceptable amount of missing data in the ovulatory period, was equal or higher than 75% (Supplementary Material).

### Users demographics

Histogram, median, and average value and standard deviations were computed for users’ age, weight, height and age at menarche when data was available. Outlier values with very low plausibility such as 45 cm for height were removed from the computations and visualization (440/13,674 (S) and 76/199,293 (K) values were removed; cycles and observations of these users were kept for the rest of the study).

### Tracking behavior

For each standard cycle, the tracking frequency was computed as the number of days with observations in that cycle divided by the length of the cycle. Cycles were labeled as with “any sexual intercourse reported” if the user logged any protected or unprotected sexual intercourse in that cycle.

### Observation description

For both app, observations of all standard cycles were summarized by cycle-day. Cycle days were either counted from the start of the cycle (first day of menstruation being day 1) or from the last day of that cycle (last day of the cycle before the next menstruation being day −1). For most tracked observations (except temperature—see below), the number of cycles for which that particular type of observation (for example “heavy” for the bleeding feature) on a specific day was divided by the total number of standard cycles for that app.

For the temperature, as the important feature to detect if ovulation has occurred is the relative rise in temperature, a reference temperature was computed for each cycle. This reference temperature was identified as the 0.25 percentile value of the temperature distribution in this cycle. Relative temperature measurements were then computed as the difference between the logged temperature and this reference temperature. The distribution (at a resolution of 0.05 °C/0.1 °F) of these relative temperatures was computed as well as the median value and the 10, 25, 75, and 90 percentile values.

### Observations decoding and ovulation timing estimation with HMM

The FAM body-signs are considered to reflect the hormonal changes orchestrating the menstrual cycles. The study was focused on understanding the extent to which these tracked cycles were consistent with previously described menstrual cycle physiologic changes, and the extent to which it was thus possible for app users to estimate timing of ovulation. Hidden Markov Models (HMM) are one of the most suitable mathematical frameworks to estimate ovulation timing, due to their ability to uncover, from observations, latent phenomenon, which in this use include the cascade of hormonal events across the menstrual cycle. HMM have also been previously used for analysis of menstrual periodicity.^20^ A 10-states HMM, in which each state is a particular phase of the menstrual cycle (Fig. 4a top, Supplementary Fig. 3A, Supplementary Material), was defined, and with decoding algorithms (Viterbi—Backward–Forward) was used to estimate the ovulation time, the uncertainty on this estimation, and a confidence score that accounts for missing observation and variation in temperature taking times.

A set of stringent criteria were established, and included: the uncertainty of the ovulation estimation (≤±1.5 days); the magnitude of the temperature shift (≥0.15 °C/0.27 °F); and the confidence score of the observations (≥0.75) to discriminate between cycles for which the estimations could be trusted (cycles with reliable ovulation estimation) and those where the observations did not allow for a reliable estimation of the ovulation day (Supplementary Fig. 4A, Supplementary Material). These strict criteria lead to the exclusion of ~40% (Sympto) and ~89% (Kindara) of the *standard cycles* that were initially selected. In total, 28,453 (Sympto) +80,708 (Kindara) cycles with reliable ovulation estimation have been used for the subsequent analyses (Supplementary Material).

### Model description

The HMM as implemented in this study describes a discretization in 10 states of the successive hormonal events throughout an ovulatory menstrual cycle. The HMM definition includes the probabilities of observing the different FAM reported body signs in each state (emission probabilities) and the probabilities of switching from one state to another (transition probabilities). Emission probabilities were chosen to reflect observations previously made in studies that tested for ovulation with LH tests or ultrasounds,^6,8,27^ while transition probabilities were chosen in a quasi-uniform manner (Supplementary Material). The ovulation estimations were robust to changes in transition probabilities but not to variations in emission probabilities (Supplementary Fig. 6, Supplementary Material), indicating that this simple framework is suitable to detect ovulations in cycles of any length, and potentially including pregnancies, relying primarily on users’ self-reported observations.

Once the model was defined, the Viterbi and the Backward–Forward algorithms^47^ were used to calculate the most probable state sequence for each cycle (Supplementary Material) and thus to estimate ovulation timing, i.e., the most likely day of the cycle in which the HMM is in the state “ovulation”. An uncertainty of the estimation has also been computed as the standard deviation of the distribution of probabilities for the state “ovulation”, which can be interpreted as the confidence interval in days for the time of ovulation estimation (Supplementary Material). Finally, a confidence score was defined to account for missing observations and variation in temperature taking time in a window of ~5 days around the estimated ovulation day (Supplementary Material).

### HMM states

The ten states, defined as a discretization of the hormonal evolution across the cycle (further details in Supplementary Material), are:

HM: Onset of the menses and the heavy/medium flow of fresh blood;

LM: Days of light bleeding or spotting that conclude menstruations;

LE: Low estrogen;

HE: High estrogen;

Ovu: Ovulation;

Rise: Temperature rise associated with rise in progesterone production;

HP: High progesterone;

EP: Estrogen peak in luteal phase;

LP: Low progesterone;

End: Artificial state for the end of each cycle.

### Reporting summary

Further information on research design is available in the Nature Research Reporting Summary linked to this article.

## Supplementary information

Supplementary Material

Reporting Summary

## Data Availability

While the privacy policies and terms of usage of the two apps (Sympto and Kindara) allow the sharing of their de-identified users’ data with third parties for research purposes but restrictions apply to the availability of these data, which were used under license for the current study, and so are not publicly available. Data are however available from the authors upon reasonable request and with permission of Sympto and Kindara. Aggregated values necessary for the production of the figures are available at https://lasy.github.io/FAM-Public-Repo/.

## Supplementary information

**Supplementary information** accompanies the paper on the *npj Digital Medicine* website (10.1038/s41746-019-0139-4).

## Code availability

The full code used for the analysis and figures presented in this study is available at https://lasy.github.io/FAM-Public-Repo/.
